# The Inhibition of the Components from Shengmai Injection towards UDP-Glucuronosyltransferase

**DOI:** 10.1155/2014/594354

**Published:** 2014-10-29

**Authors:** Li-Peng Jiang, Jin Zhao, Yun-Feng Cao, Mo Hong, Dong-Xue Sun, Xiao-Yu Sun, Jun Yin, Zhi-Tu Zhu, Zhong-Ze Fang

**Affiliations:** ^1^The First Affiliated Hospital of Liaoning Medical University, Jinzhou, Liaoning 121001, China; ^2^School of Traditional Chinese Medicine, Shenyang Pharmaceutical University, Shenyang 110016, China; ^3^Key Laboratory of Contraceptives and Devices Research (NPFPC), Shanghai Engineer and Technology Research Center of Reproductive Health Drug and Devices, Shanghai Institute of Planned Parenthood Research, Shanghai 200032, China; ^4^Joint Center for Translational Medicine, Dalian Institute of Chemical Physics, Chinese Academy of Sciences and The First Affiliated Hospital of Liaoning Medical University, No. 457, Zhongshan Road, Dalian 116023, China; ^5^Joint Center for Translational Medicine, Dalian Institute of Chemical Physics, Chinese Academy of Sciences and The Affiliated Zhongshan Hospital of Dalian University, No. 6, Jiefang Street, Zhongshan District, Dalian 116001, China; ^6^Department of Toxicology, School of Public Health, Tianjin Medical University, 22 Qixiangtai Road, Heping District, Tianjin 300070, China

## Abstract

The mechanism of shengmai injection- (SMI-) related drug-drug interaction remains unclear. Evaluation of the inhibition potential of SMI's ingredients towards UDP-glucuronosyltransferases (UGTs) activity will provide a new insight to understand SMI-related drug-drug interaction. *In vitro* incubation system to model UGT reaction was used. Recombinant UGT isoforms-catalyzed 4-methylumbelliferone (4-MU) glucuronidation and UGT1A4-catalyzed trifluoperazine (TFP) glucuronidation reactions were employed to phenotype the inhibition profile of maidong's components towards the activity of UGT isoforms. Different inhibition potential of maidong's components towards various UGT isoforms was observed. Based on the inhibition kinetic investigation results, ophiopogonin D (OD) noncompetitively inhibited UGT1A6 and competitively inhibited UGT1A8, ophiopogonin D′ (OD′) noncompetitively inhibited UGT1A6 and UGT1A10, and ruscorectal (RU) exhibited competitive inhibition towards UGT1A4. The inhibition kinetic parameters were calculated to be 20.6, 40.1, 5.3, 9.0, and 0.02 *μ*M, respectively. In combination with our previous results obtained for the inhibition of UGT isoforms by ginsenosides and wuweizi components, the important SMI ingredients exhibiting strong inhibition towards UGT isoforms were highlighted. All the results obtained in the present study provide a new insight to understand SMI-related drug-drug interaction.

## 1. Introduction

Shengmai San, a well-known traditional Chinese herbal prescription recorded in Yixueqiyuan (Origins of Medicine), has been routinely employed to treat cardiovascular diseases for thousands of years in China [1]. Shengmai injection (SMI), developed on the basis of Shengmai San, has been clinically applied. SMI contains the following herbs: Renshen (Radix Ginseng; Ginseng), Maidong (Radix Ophiopogonis; Dwarf Lilyturf Tuber), and Wuweizi (Fructus schisandrae* chinensis*; Chinese Magnoliavine Fruit). The clinical safety of SMI has been speculated, and the adverse effects were detected in 36 patients taking SMI, including 17 male and 19 female [2]. The main adverse effect for patients taking SMI is anaphylactic reaction [2]. Additionally, clinical drug-drug interaction between SMI and warfarin was also reported [3].

UDP-glucuronosyltransferases (UGTs) are important metabolizing enzymes involved in the metabolism of many xenobiotics (e.g., propofol, zidovudine, etc.) and endogenous substances (e.g., bilirubin, bile acids, etc.) [4]. UGTs have been demonstrated to exhibit expression in multiple tissues, mainly in liver and intestine. To date, approximately 117 members have been found in mammalian UGT gene superfamily, and they are divided into four families, UGT1, UGT2, UGT3, and UGT8. Inhibition of UGTs' activity can induce severe adverse effects. Indinavir, an important HIV therapeutic drug, has been reported to significantly induce the elevation of serum-unconjugated bilirubin through inhibiting UGT1A1-catalyzed bilirubin glucuronidation [5]. Sorafenib-induced UGT1A1 inhibition might also be an important reason for the elevation of serum bilirubin [6].

To date, the mechanism for SMI-related drug-drug interaction remains unclear. Evaluation of SMI's components' inhibitory potential towards UGTs isoforms will provide us with a new insight to elucidate the mechanism of SMI-related drug-drug interaction. In our previous study, relevant work has been performed for ginseng and wuweizi's ingredients. Ginsenosides, the major components of ginseng, have been demonstrated to exhibit strong structure-dependent inhibition towards many UGT isoforms [7]. Additionally, deoxyschizandrin and schisantherin A, two important ingredients isolated from wuweizi, have been demonstrated to exhibit inhibition towards UGT1A3 [8].

All these results give us the data support and confidence to investigate the inhibition of maidong's components towards UGT isoforms. Using the combination of the present data and previous UGTs' inhibition data for ginsenosides and wuweizi lignans, the important SMI ingredients exhibiting strong inhibition towards UGT isoforms were highlighted.

## 2. Materials and Methods

### 2.1. Chemicals

4-Methylumbelliferone (4-MU), 4-methylumbelliferone-*β*-D-glucuronide (4-MUG), Tris-HCl, 7-hydroxycoumarin, and uridine-5′-diphosphoglucuronic acid (UDPGA) (trisodium salt) were purchased from Sigma-Aldrich (St. Louis, MO). Recombinant human UGT isoforms (UGT1A1, UGT1A3, UGT1A4, UGT1A6, UGT1A7, UGT1A8, UGT1A9, UGT1A10, UGT2B4, UGT2B7, and UGT2B15) expressed in baculovirus-infected insect cells were obtained from BD Gentest Corp. (Woburn, MA, USA). Ophiopogonin D (OD), ophiopogonin D′ (OD′), methylophiopogonanone A (MA), methylophiopogonanone B (MB), and ruscorectal (RU) were purchased from Sichuan Weikeqi Biotechnology Company (Chengdu, Sichuan, China). The purity of these compounds was above 95%. All other reagents were of HPLC grade or of the highest grade commercially available.

### 2.2. Initial Screening of Maidong' Components Inhibition towards Recombinant UGTs Activity

4-MU, the nonspecific probe substrate for all the UGT isoforms except UGT1A4, was used in the present study to initially screen the inhibition potential of maidong's components towards UGT isoforms as previously reported [7]. In brief, the incubation system (total volume = 200 *μ*L) consisted of recombinant UGTs, 5 mM UDPGA, 5 mM MgCl_2_, 50 mM Tris-HCl (pH = 7.4), and 4-MU in the absence or presence of different concentrations of maidong's components. The compounds were dissolved in DMSO, and the final concentration of DMSO was below 0.5% (v/v). The used incubation time and protein concentration were previously determined to ensure the reaction rate within the linear range. The 4-MU concentration was equal to known *K*_*m*_ or *S*_50_ values for each UGT isoform [7]. After 5 min preincubation at 37°C, the incubation reaction was initiated via adding UDPGA to the mixture system. The reactions were quenched by adding 100 *μ*L acetonitrile with 7-hydroxycoumarin (100 *μ*M) as internal standard. Centrifuged at 20000 ×g for 10 min, an aliquot of supernatant was transferred to an autoinjector vial for HPLC analysis. The HPLC system (Shimadzu, Kyoto, Japan) contained a SCL-10A system controller, two LC-10AT pumps, a SIL-10A autoinjector, and a SPD-10AVP UV detector. Chromatographic separation was carried out using a C18 column (4.6 × 200 mm, 5 *μ*m, Kromasil) at a flow rate of 1 mL/min and UV detector at 316 nm. The mobile phase consisted of acetonitrile (A) and H_2_O containing 0.5% (v/v) formic acid (B). The following gradient condition was used: 0–15 min, 95–40% B; 15–20 min, 10% B; 20–30 min, 95% B.

UGT1A4 exhibited low catalytic activity towards 4-MU glucuronidation. Therefore, UGT1A4-catalyzed trifluoperazine (TFP) glucuronidation was carried out to evaluate inhibition of maidong's components towards UGT1A4 activity. As previously reported [9], TFP (40 *μ*M, near its *K*_*m*_ value) was incubated with 0.1 mg/mL recombinant UGT1A4 for 20 min in the presence or absence of maidong's ingredients.

### 2.3. Inhibition Kinetic Analysis

To determine the inhibition kinetic type and parameters (*K*_*i*_), the reaction velocity (*V*) was determined at multiple concentrations of maidong's components and 4-MU (or TFP). Dixon plot and Lineweaver-Burk plot were used to fit the data. The inhibition kinetic type was evaluated through determining the intersection point in the Dixon and Lineweaver-Burk plots.

Nonlinear regression was used to calculate the inhibition kinetic parameters (*K*_*i*_) according to the equations for competitive inhibition (1) and noncompetitive inhibition (2):

(1) $$\begin{array}{c} V=\frac{V_{\max}*\left[S\right]}{\left(K_{m}/\left(1+\left(\left[I\right]/K_{i}\right)\right)+\left[S\right]\right)}, \end{array}$$

(2) $$\begin{array}{c} V=\frac{V_{\max}*\left[S\right]}{\left(K_{m}+\left[S\right]\right)*\left(1+\left(\left[I\right]/K_{i}\right)\right)}, \end{array}$$

where the items are defined as follows: *V* is the reaction velocity and [*S*] and [*I*] are the concentrations of substrate and inhibitor, respectively. *K*_*m*_ value is the substrate concentration in which the velocity reached half of the maximum velocity (*V*_max⁡_) of the reaction. *K*_*i*_ value is the inhibition constant.

## 3. Results

### 3.1. Differential Inhibitory Potential of Maidong's Components towards Different UGT Isoforms

**Figure 1:**
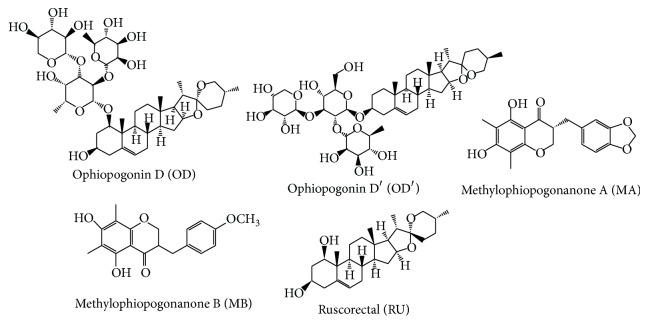
The structures of ophiopogonin D (OD), ophiopogonin D′ (OD′), methylophiopogonanone A (MA), methylophiopogonanone B (MB), and ruscorectal (RU).

The structures of maidong's components were shown in Figure 1, including ophiopogonin D (OD), ophiopogonin D′ (OD′), methylophiopogonanone A (MA), methylophiopogonanone B (MB), and ruscorectal (RU). The different structure of all these compounds resulted in the different inhibition potential towards UGT isoforms (Table 1). All the compounds showed little influence towards the activity of UGT1A1, 1A3, 1A7, 1A9, 1A10, 2B4, 2B7, and 2B15, with the inhibition percent less than 20% at 100 *μ*M. RU exhibits the strongest inhibition towards UGT1A4, with almost 100% inhibition at 100 *μ*M of RU. 100 *μ*M of OD and OD′ exerted strong inhibition towards UGT1A6 (87.2%) and UGT1A8 (82.7%). The activity of UGT1A6 was also strongly inhibited by OD′, with the activity inhibited by 87.9% at 100 *μ*M of OD′. The activity of UGT1A10 was inhibited by 80.2% by 100 *μ*M of OD′.

### 3.2. Inhibition Type and Parameters of Maidong's Components towards Representative UGT Isoforms

**Figure 2:**
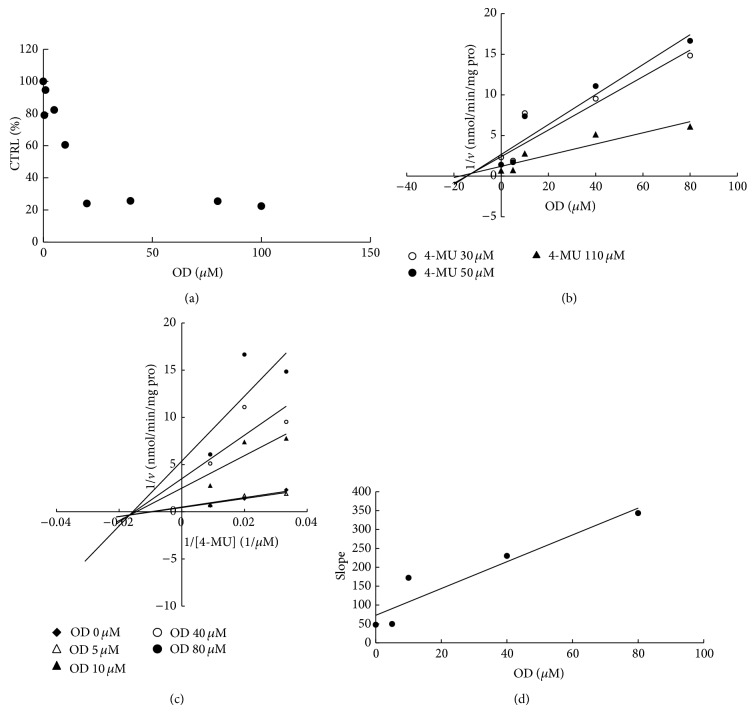
Determination of inhibition type and parameters (*K*_*i*_) of OD towards UGT1A6-catalyzed 4-MU glucuronidation reaction. (a) Dose-dependent inhibition of OD towards UGT1A6-catalyzed 4-MU glucuronidation. (b) Dixon plot of inhibition of OD towards UGT1A6-catalyzed 4-MU glucuronidation. (c) Lineweaver-Burk plot of inhibition of OD towards UGT1A6-catalyzed 4-MU glucuronidation. (d) Second plot of inhibition of OD towards UGT1A6-catalyzed 4-MU glucuronidation. The data point represents the mean value of duplicate experiments.

**Figure 3:**
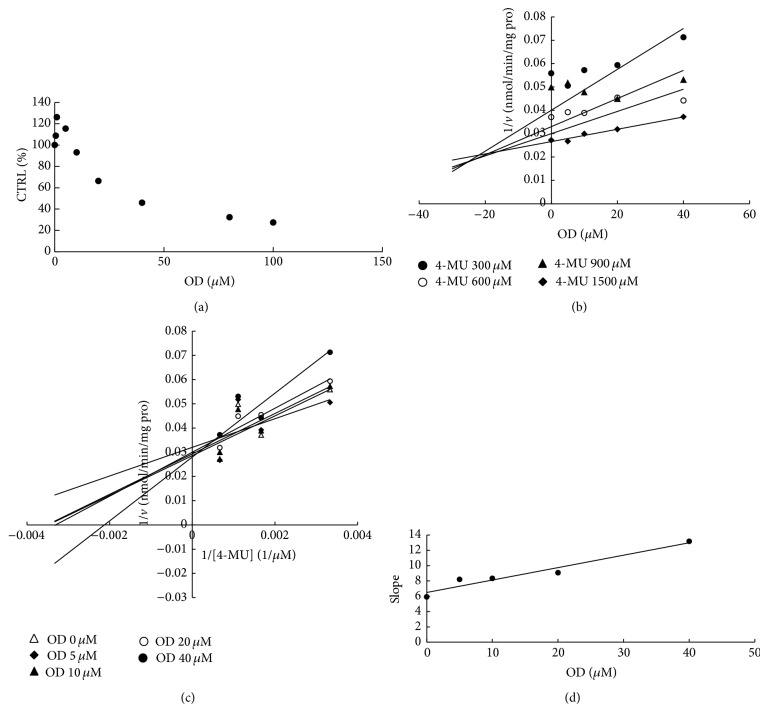
Determination of inhibition type and parameters (*K*_*i*_) of OD towards UGT1A8-catalyzed 4-MU glucuronidation reaction. (a) Dose-dependent inhibition of OD towards UGT1A8-catalyzed 4-MU glucuronidation. (b) Dixon plot of inhibition of OD towards UGT1A8-catalyzed 4-MU glucuronidation. (c) Lineweaver-Burk plot of inhibition of OD towards UGT1A8-catalyzed 4-MU glucuronidation. (d) Second plot of inhibition of OD towards UGT1A8-catalyzed 4-MU glucuronidation. The data point represents the mean value of duplicate experiments.

**Figure 4:**
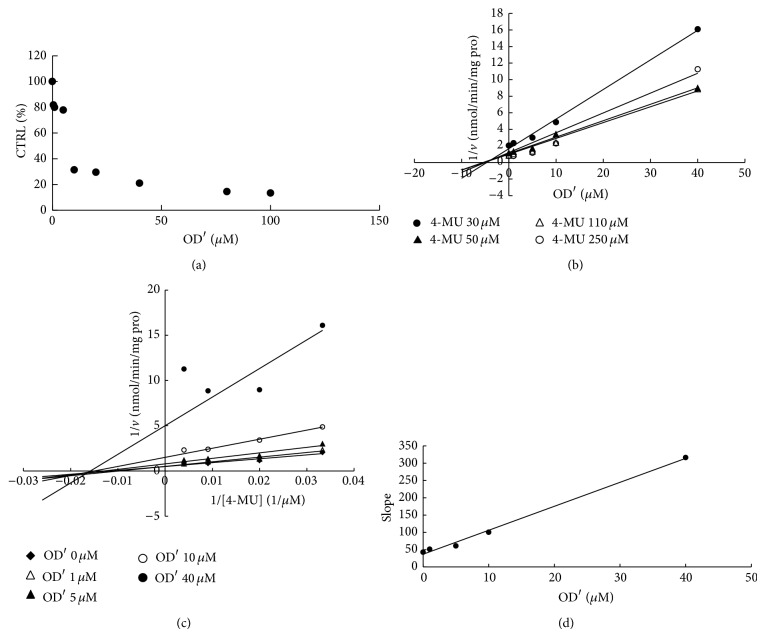
Determination of inhibition type and parameters (*K*_*i*_) of OD′ towards UGT1A6-catalyzed 4-MU glucuronidation reaction. (a) Dose-dependent inhibition of OD′ towards UGT1A6-catalyzed 4-MU glucuronidation. (b) Dixon plot of inhibition of OD′ towards UGT1A6-catalyzed 4-MU glucuronidation. (c) Lineweaver-Burk plot of inhibition of OD′ towards UGT1A6-catalyzed 4-MU glucuronidation. (d) Second plot of inhibition of OD′ towards UGT1A6-catalyzed 4-MU glucuronidation. The data point represents the mean value of duplicate experiments.

**Figure 5:**
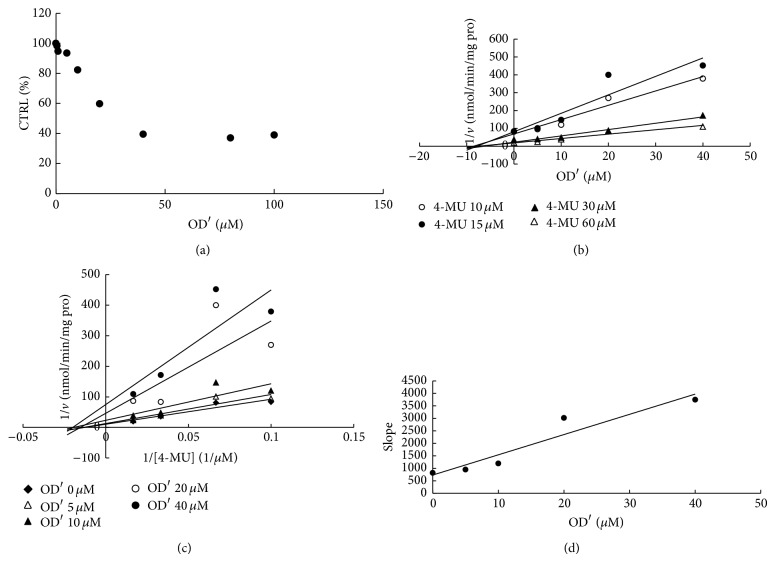
Determination of inhibition type and parameters (*K*_*i*_) of OD′ towards UGT1A10-catalyzed 4-MU glucuronidation reaction. (a) Dose-dependent inhibition of OD′ towards UGT1A10-catalyzed 4-MU glucuronidation. (b) Dixon plot of inhibition of OD′ towards UGT1A10-catalyzed 4-MU glucuronidation. (c) Lineweaver-Burk plot of inhibition of OD′ towards UGT1A10-catalyzed 4-MU glucuronidation. (d) Second plot of inhibition of OD′ towards UGT1A10-catalyzed 4-MU glucuronidation. The data point represents the mean value of duplicate experiments.

**Figure 6:**
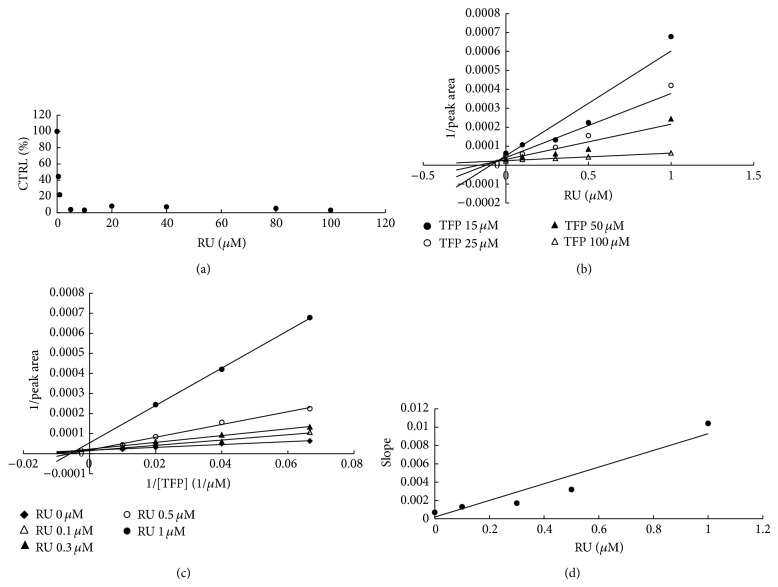
Determination of inhibition type and parameters (*K*_*i*_) of RU towards UGT1A4-catalyzed TFP glucuronidation reaction. (a) Dose-dependent inhibition of RU towards UGT1A4-catalyzed TFP glucuronidation. (b) Dixon plot of inhibition of RU towards UGT1A4-catalyzed TFP glucuronidation. (c) Lineweaver-Burk plot of inhibition of RU towards UGT1A4-catalyzed TFP glucuronidation. (d) Second plot of RU's inhibition towards UGT1A4-catalyzed TFP glucuronidation. The data point represents the mean value of duplicate experiments.

To furtherly obtain the inhibition kinetic information, we evaluated the inhibition type and parameters of representative inhibition of compounds towards UGT isoforms. The concentration-dependent inhibition was evaluated, and Dixon plot and Lineweaver-Burk plot were employed for elucidation of the inhibition type. Dose-dependent inhibition behaviour can be observed for the inhibition of OD towards UGT1A6 and UGT1A8 (Figures 2(a) and 3(a)), the inhibition of OD′ towards UGT1A6 and UGT1A10 (Figures 4(a) and 5(a)), and the inhibition of RU towards UGT1A4 (Figure 6(a)). OD noncompetitively inhibited UGT1A6 and competitively inhibited UGT1A8, as demonstrated by Dixon plot (Figures 2(b) and 3(b)) and Lineweaver-Burk plot (Figures 2(c) and 3(c)). OD′ noncompetitively inhibited UGT1A6 and UGT1A10 (Figures 4(b), 4(c), 5(b), and 5(c)). Dixon plot (Figure 6(b)) and Lineweaver-Burk plot (Figure 6(c)) showed that RU exerted competitive inhibition towards UGT1A4-catalyzed TFP glucuronidation reaction. The inhibition kinetic parameters (*K*_*i*_) were calculated to be 20.6, 40.1, 5.3, 9.0, and 0.02 *μ*M for the inhibition of OD towards UGT1A6 and UGT1A8 (Figures 2(d) and 3(d)), the inhibition of OD′ towards UGT1A6 and UGT1A10 (Figures 4(d) and 5(d)), and the inhibition of RU towards UGT1A4 (Figure 6(d)).

### 3.3. Highlight of Components in SMI Showing Strong Inhibition towards UGT Isoforms

**Figure 7:**
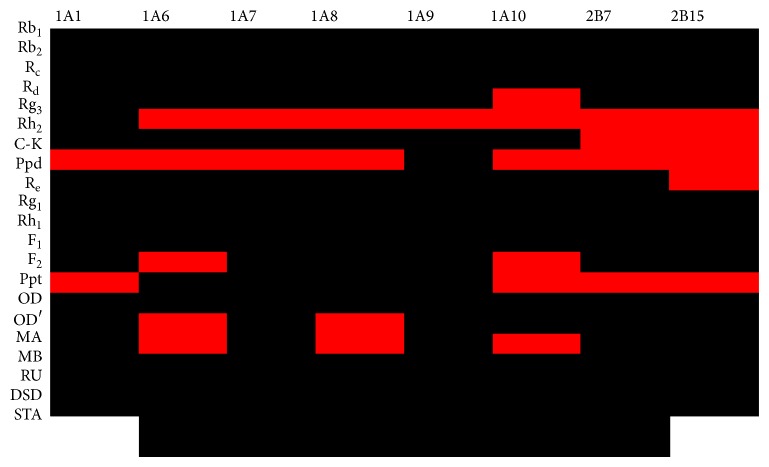
Highlight of components in SMI showing strong inhibition towards UGT isoforms. The residual activity below 20% at 100 *μ*M was highlighted with red color, and the residual activity above 20% at 100 *μ*M was highlighted with black color.

We made a relatively complete description on the inhibition potential of SMI major components towards UGT isoforms, including ginsenosides and maidong's and wuweizi's ingredients (Figure 7). The residual activity below 20% at 100 *μ*M was highlighted with red color, and the residual activity above 20% at 100 *μ*M was highlighted with black color. This figure can clearly tell the readers which components mainly contributed to the inhibition of SMI towards UGT activity.

## 4. Discussion

The safety problem of herbs should be paid much attention, especially when the administration route changes from oral administration to injection because the detoxification function of intestine is avoided. Although searching the targets and substances basis for herbal toxicity is a tremendous job, our previous results encourage our recent study to evaluate the inhibition potential of maidong's major components towards UGT isoforms supposing that UGTs are potential toxicity targets of shengmai injection (SMI).

The information obtained in the present study can help us to obtain a relatively complete inhibition profile of SMI major components towards important UGT isoforms. As is expected, the ingredients of maidong can show the inhibitory potential towards some UGT isoforms, such as the inhibition of UGT1A6 and UGT1A8 by OD, the inhibition of UGT1A6 and UGT1A10 by OD′, and the inhibition of UGT1A4 by RU. From the complete inhibitory profile of SMI ingredients towards UGT isoforms, ginsenosides Rg_3_ and C-K are the top two components influencing most UGT isoforms. Additionally, ginsenosides R_d_, F_2_, ppt, and maidong's components OD and OD′ also significantly contributed to the UGT inhibition by SMI. UGT isoforms play key roles in the metabolism of many endogenous substances. For example, UGT1A1 can catalyze the conjugation reaction of bilirubin [5, 6]. UGT1A3, UGT1A4, and UGT2B7 can conjugate the bile acids, androgens, and estrogens components through glucuronidation reactions [10, 11]. Therefore, the influence towards the activity of these UGT isoforms might result in the metabolic disorders of these substances, which might be a potential reason for SMI-induced adverse effects. The* in vivo* inhibition situation was not performed for SMI due to the little information on the quantity of individual ingredients and the exposure level of these components in plasma.

Many complex factors might affect the explanation of these* in vitro* data. For example, the activation of drug-metabolizing enzymes (DMEs) through nuclear receptor (e.g., pregnane X receptor (PXR), peroxisome proliferator-activated receptor (PPAR), aryl hydrocarbon receptor (Ahr), etc.) is an important reason. Ginsenosides F_2_ and ppt were demonstrated to exhibit moderate inductive potential towards PXR [12]. The experiment performed by Lee et al. showed that ginsenoside R_f_ can regulate lipoprotein metabolism through affecting the activity of PPARalpha [13]. Ginsenosides were also demonstrated to be naturally occurring aryl hydrocarbon receptor (ahr) ligands [14]. Wuweizi ingredients have also been demonstrated to be strong activators towards PXR [15]. All these affected nuclear receptors have been indicated to be able to induce the expression of UGT isoforms [16], which might complicate the explanation of the* in vitro* inhibition data. Another important factor affecting the explanation of* in vitro* parameters is the noncomplete understanding of the constitution of SMI. Although numerous high-grade technologies have been used to elucidate the components, the global characterization of ingredients contained in SMI remains to be challenged [17]. Therefore, all the unknown components in SMI might play an unexpected role towards UGT isoforms-mediated reactions.

In conclusion, the inhibition capability of major components of maidong towards important UGT isoforms was investigated to make relatively complete inhibition profile of SMI ingredients towards the important UGTs. Although many complicated factors might influence the translation of these* in vitro* results into* in vivo* situation, all the information obtained in the present study provides a new insight to understand shengmai injection- (SMI-) related drug-drug interaction.

## Supplementary Material

The representative chromatography spectrum of 4-MU incubation sample was given in Supplemental Figure 1. The retention time was 2.6 min, 3.1 min, and 3.6 min for 4-MUG, internal standard, and 4-MU, respectively.

## Figures and Tables

**Table 1:** Initial screening of the inhibitory effects of ophiopogonin D (OD), ophiopogonin D′ (OD′), methylophiopogonanone A (MA), methylophiopogonanone A (MB), and ruscorectal (RU). 100 *μ*M of compounds was selected. The values shown in this table are residual activity which can be calculated using the following equation: % residual activity = the activity at 100 *μ*M of compounds/the activity at 0 *μ*M of compounds.

	OD	OD′	MA	MB	RU
UGT1A1	77.1 ± 3.8	81.0 ± 12.6	31.3 ± 1.3	27.9 ± 11.1	41.5 ± 36.9
UGT1A3	40.7 ± 0.3	36.5 ± 2.5	35.5 ± 6.1	84.2 ± 22.8	37.2 ± 18.2
UGT1A4	24.6 ± 1.6	21.0 ± 1.6	70.4 ± 3.8	81.1 ± 8.0	0.0 ± 0.0
UGT1A6	12.8 ± 3.1	12.1 ± 1.9	67.0 ± 1.7	65.3 ± 2.0	65.9 ± 11.6
UGT1A7	143.5 ± 8.7	122.2 ± 1.3	57.5 ± 10.8	83.4 ± 5.9	52.4 ± 7.4
UGT1A8	17.3 ± 0.9	18.9 ± 6.6	20.5 ± 2.1	85.0 ± 4.5	72.0 ± 3.0
UGT1A9	135.3 ± 1.5	133.8 ± 2.1	38.7 ± 0.0	68.2 ± 1.5	110.7 ± 1.1
UGT1A10	26.5 ± 1.5	19.8 ± 3.2	70.0 ± 9.3	96.9 ± 7.3	82.0 ± 5.2
UGT2B4	101.7 ± 2.6	118.0 ± 21.8	71.1 ± 1.5	73.2 ± 1.2	88.4 ± 1.6
UGT2B7	85.4 ± 7.3	77.4 ± 7.1	23.3 ± 16.7	82.8 ± 14.5	124.8 ± 59.0
UGT2B15	36.9 ± 0.0	35.5 ± 1.7	23.8 ± 2.8	56.1 ± 9.8	55.5 ± 19.6
