# Supplementary material for: The Inhibition of the Components from Shengmai Injection towards UDP-Glucuronosyltransferase
Source: Evid Based Complement Alternat Med. 2014 Oct 29;2014:594354. doi: 10.1155/2014/594354 (PMC4229968; doi:10.1155/2014/594354)
Supplement: Supplementary file 1 — The representative chromatography spectrum of 4-MU incubation sample was given in Supplemental Figure 1. The retention time was 2.6 min, 3.1 min, and 3.6 min for 4-MUG, internal standard, and 4-MU, respectively. [file 594354.f1.doc]

**Supplemental Materials:**

**Supplemental Figure 1: Representative Chromatography Spectrum of 4-MU incubation sample. The retention time was 2.6 min, 3.1 min, and 3.6 min for 4-MUG, I.S., and 4-MU, respectively.**
